# Supplementary material for: Deep transfer learning of cancer drug responses by integrating bulk and single-cell RNA-seq data
Source: Nat Commun. 2022 Oct 30;13:6494. doi: 10.1038/s41467-022-34277-7 (PMC9618578; doi:10.1038/s41467-022-34277-7)
Supplement: Supplementary file 1 — Supplementary Information [file 41467_2022_34277_MOESM1_ESM.docx]

**Supplementary Information of**

**Deep Transfer Learning of Cancer Drug Responses by Integrating Bulk and Single-cell RNA-seq data**

Junyi Chen^1,*^, Xiaoying Wang^2,*^, Anjun Ma^1,3,$^, Qi-En Wang^4^, Bingqiang Liu^2^, Lang Li^1^, Dong Xu^5^, Qin Ma^1,3,$^

^1^ Department of Biomedical Informatics, College of Medicine, The Ohio State University, Columbus, OH 43210, USA

^2^ Department of Mathematics, Shandong University, Shandong 250100, China

^3^ Pelotonia Institute for Immuno-Oncology, The James Comprehensive Cancer Center, The Ohio State University, Columbus, OH 43210, USA.

^4^ Department of Radiation Oncology, Comprehensive Cancer Center, The Ohio State University, Columbus, OH 43210, USA

^5^ Department of Electrical Engineering and Computer Science, and Christopher S. Bond Life Sciences Center, University of Missouri, Columbia, MO 65211, USA

^*^ These authors contributed equally

^$^ To whom correspondence should be addressed:

Qin Ma: [qin.ma@osumc.edu](mailto:qin.ma@osumc.edu)

Anjun Ma: [anjun.ma@osumc.edu](mailto:anjun.ma@osumc.edu).

Supplementary Table S1. Summary of the scRNA-seq datasets.

|  | **Authors** | **Drug** | **GEO access** | **Cells** | **Species** | **Cancer type** |
| --- | --- | --- | --- | --- | --- | --- |
| Data 1 | Sharma, et al. | Cisplatin | GSE117872 | 548 | Homo sapiens | Oral squamous cell carcinomas |
| Data 2 |  |  |  | 568 |  |  |
| Data 3 | Kong, et al. | Gefitinib | GSE112274 | 507 | Homo sapiens | Lung cancer |
| Data 4 | Schnepp, et al. | Docetaxel | GSE140440 | 324 | Homo sapiens | Prostate Cancer |
| Data 5 | Aissa, et al. | Erlotinib | GSE149383 | 1496 | Homo sapiens | Lung cancer |
| Data 6 | Bell, et al. | I-BET-762 | GSE110894 | 1419 | Mus musculus | Acute myeloid leukemia |

**Supplementary Table S2.** Optimized parameters in training autoencoder for bulk and single-cell feature extraction.

| **Drug** | **Sampling** | **Bottle-neck** | **Encoder**  **hidden dimensions** | **Predictor hidden**  **dimension** |
| --- | --- | --- | --- | --- |
| Cisplatin | Up-sampling | 64 | (512, 256) | (32, 16) |
| Cisplatin | Up-sampling | 128 | (512, 256) | (64, 32) |
| Gefitinib | Down-sampling | 256 | (256, 256) | (128, 64) |
| Docetaxel | SMOTE-sampling | 100 | (512, 256) | (64, 32) |
| Erlotinib | Downs-sampling | 128 | (512, 256) | (64, 32) |
| I-BET-762 | Down-sampling | 256 | (256, 256) | (128, 64) |

Supplementary Table S3. Classification results of testing subset of bulk RNA-seq data.

| **Drug** | **Precision** | **Recall** | **F1-score** | **AUROC** | **AP score** |
| --- | --- | --- | --- | --- | --- |
| Cisplatin (for Data 1) | 0.870 | 0.860 | 0.862 | 0.916 | 0.834 |
| Cisplatin (for Data 2) | 0.888 | 0.868 | 0.871 | 0.925 | 0.823 |
| Gefitinib | 0.885 | 0.881 | 0.878 | 0.955 | 0.929 |
| Docetaxel | 0.961 | 0.961 | 0.961 | 0.965 | 0.950 |
| Erlotinib | 0.928 | 0.927 | 0.927 | 0.964 | 0.918 |
| I-BET-762 | 0.929 | 0.929 | 0.929 | 0.966 | 0.942 |
| **Mean** | 0.910 | 0.904 | 0.905 | 0.949 | 0.899 |
| **SD** | 0.032 | 0.037 | 0.036 | 0.020 | 0.051 |

**Supplementary Table S4.** Overview information of GDSC and CCEL databases.

|  | # cell lines | # drugs | # genes |
| --- | --- | --- | --- |
| GDSC | 804 | 192 | 17,419 |
| CCLE | 476 | 1448 | 19,177 |
| GDSC+CCLE | 1280 | 1557 | 15,962 |

**Supplementary Table S5.** Number of cell lines included in the bulk databases that have drug response files for drugs in our benchmarking data.

| **Drugs** | **GDSC** | **CCLE** | **GDSC+CCLE** |
| --- | --- | --- | --- |
| Cisplatin | 765 | 443 | 1208 |
| Gefitinib | 749 | 463 | 1212 |
| Docetaxel | 799 | 474 | 1273 |
| Erlotinib | 749 | 474 | 1223 |
| I-BET-762 | 725 | 467 | 1192 |

**Supplementary Table S6.** Number of genes showing positive IG scores to bulk level and single-cell level drug responses.

|  | For drug sensitivity | | | For drug resistance | | |
| --- | --- | --- | --- | --- | --- | --- |
|  | contributing genes # at bulk level | contributing genes # at single-cell level | % of overlaps | contributing genes # at bulk level | contributing genes # at single-cell level | % of overlaps |
| Data 1 | 1587 | 1294 | 55% | 1600 | 1712 | 57% |
| Data 2 | 1675 | 1828 | 41% | 1668 | 1404 | 83% |
| Data 3 | 1784 | 1858 | 48% | 1439 | 1187 | 48% |
| Data 4 | 900 | 1173 | 49% | 1153 | 1238 | 49% |
| Data 5 | 1890 | 1842 | 33% | 1027 | 1073 | 32% |
| Data 6 | 1853 | 1807 | 48% | 1241 | 1248 | 50% |


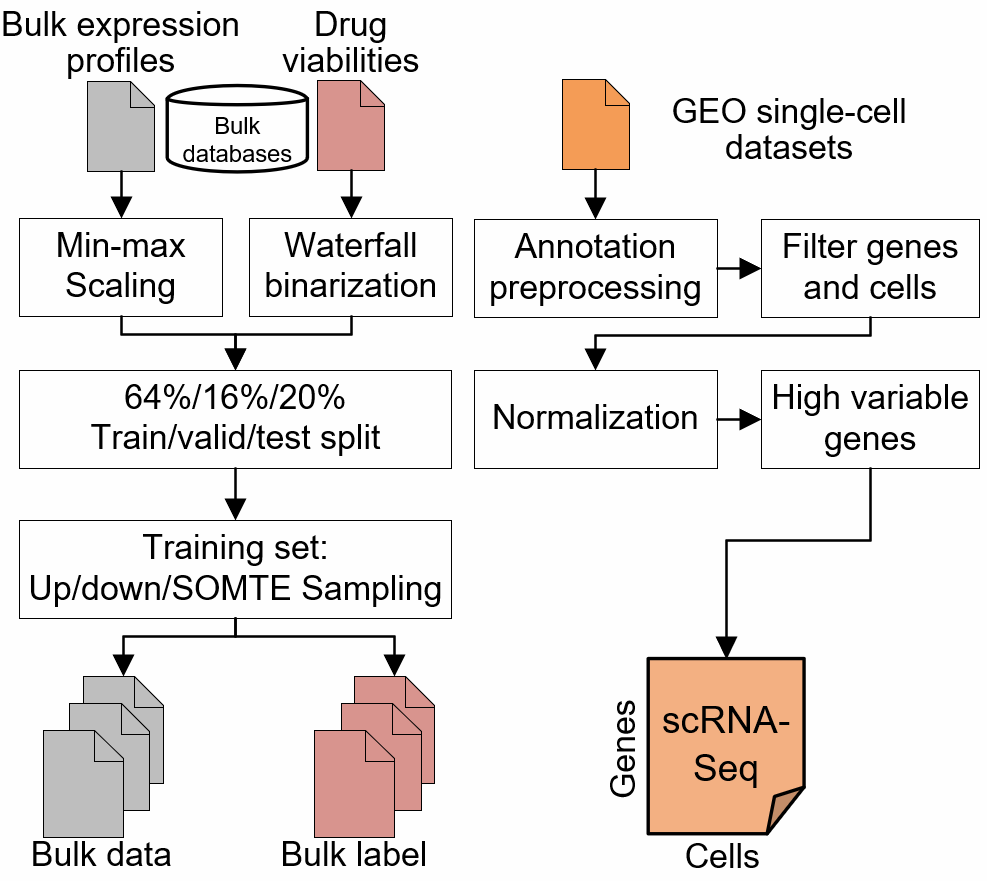
Supplementary Figure S1. The preprocessing workflow used for bulk RNA-seq and scRNA-seq data before scDEAL model training.


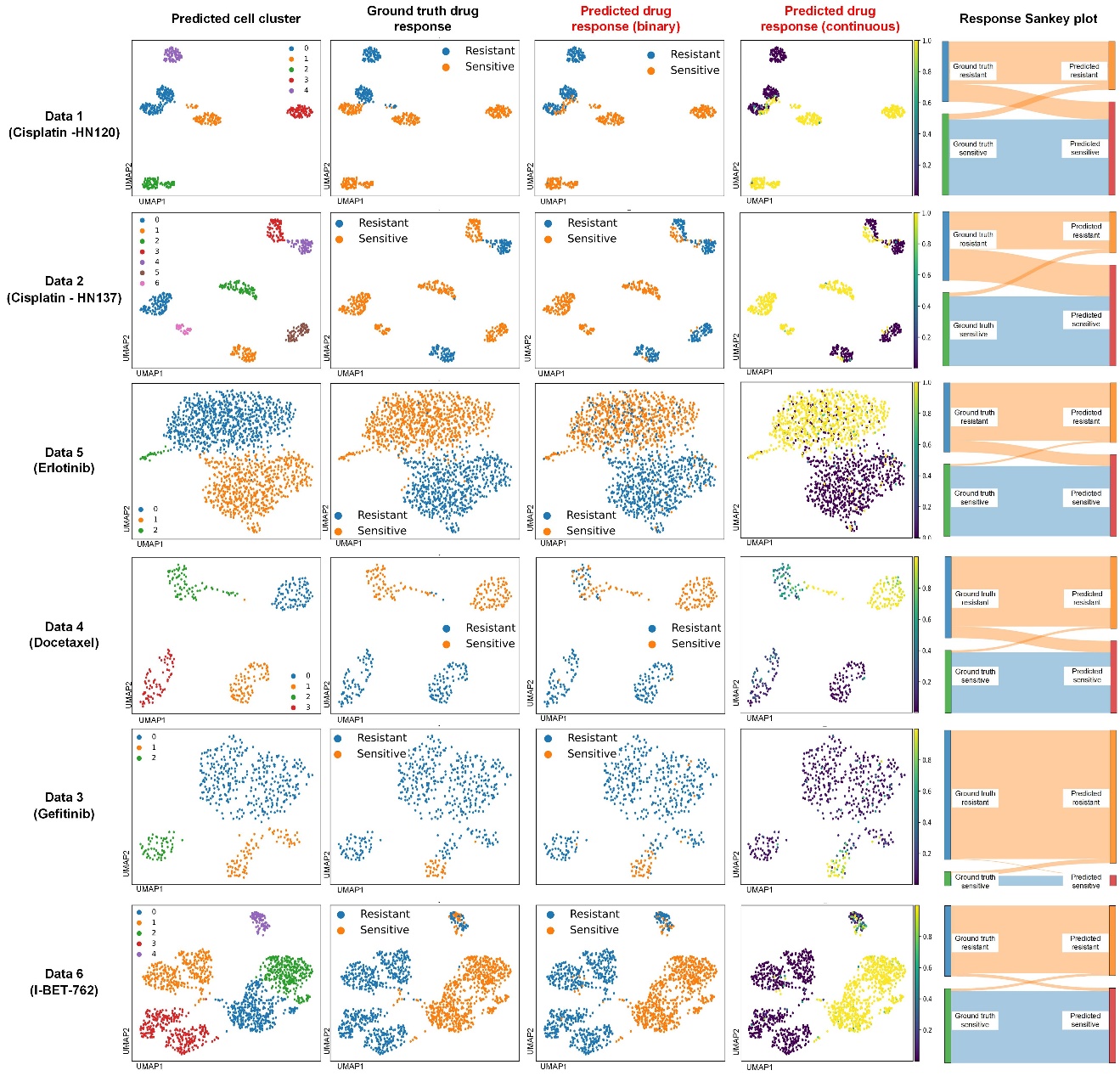
**Supplementary Figure S2**. **Benchmark results of six datasets with optimized parameters.** UMAPs were generated for each dataset and colored them by predicted cell clusters, ground- truth single-cell drug responses, scDEAL-predicted drug responses (binary labels as well as continuous probability), and generated Sankey plots to observe the discrepancies between the ground-truth and predicted labels.


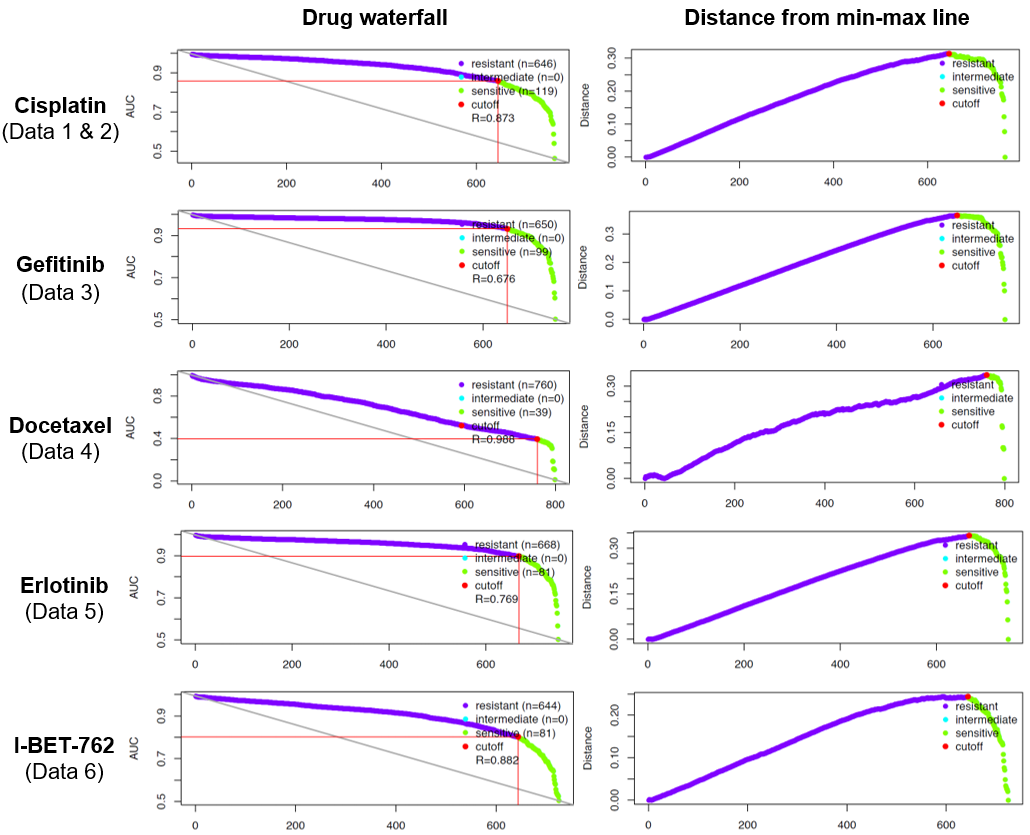


Supplementary Figure S3. Waterfall plots and min-max distance plots of bulk drug responses. The binarization on drug response profiles of all bulk cell lines for the five selected drugs are showcased. The X-axis of each waterfall plot is the rank of cell line AUC values in descending order. The Y-axis of the left panel is the AUC value, and the Y-axis in the right panel is the distance to the line cell lines having the largest and the lowest AUC values. The red dots represent the cutoff of AUC values. Cell lines having AUC lower than the cutoff are marked as resistant and vice versa.


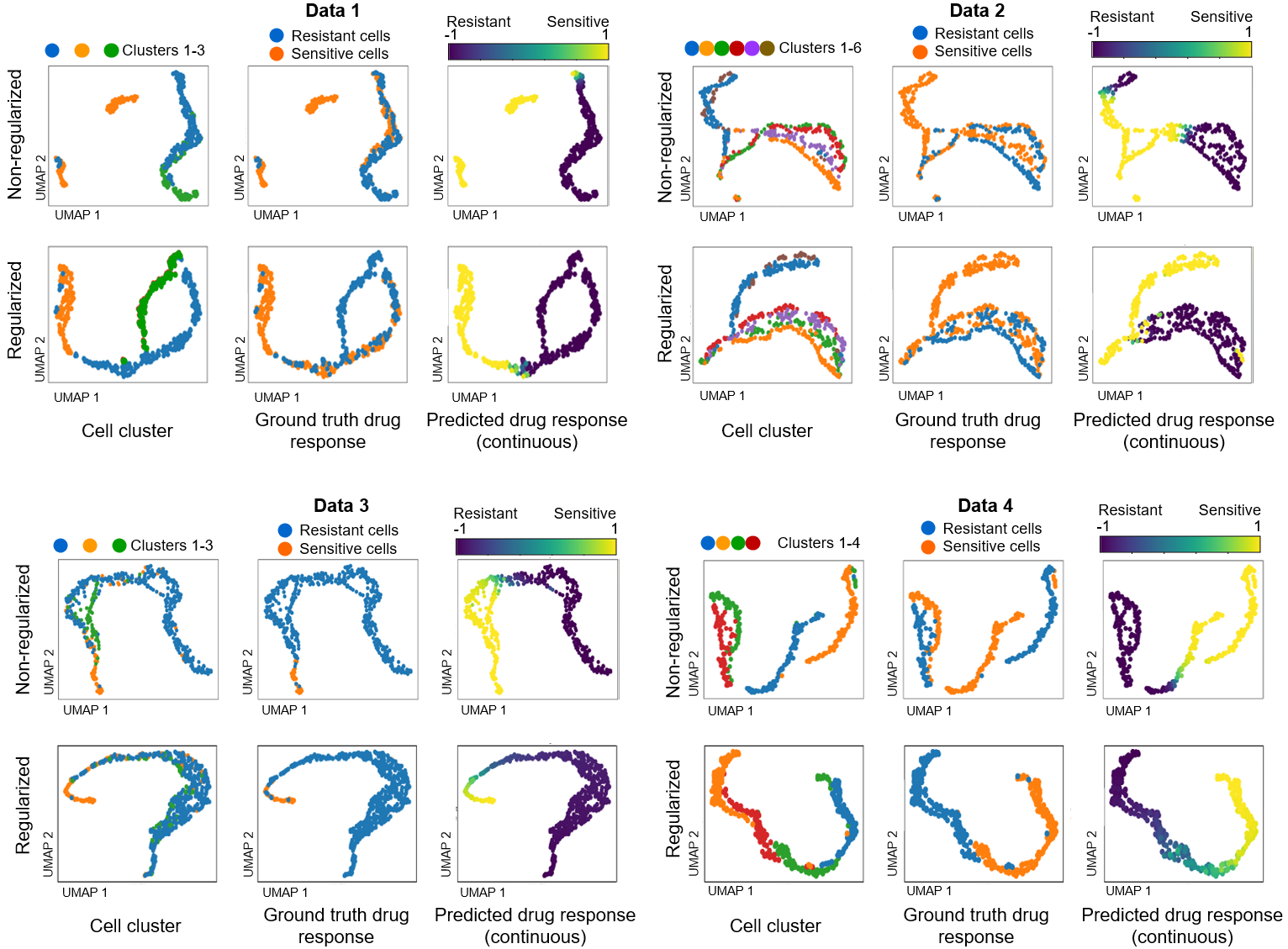


**Supplementary Figure S4**. **UMAPs of Data 1-4 with and without adding cell type regularization.** were generated directly using scDEAL latent representations of single cells corresponding to drug response.


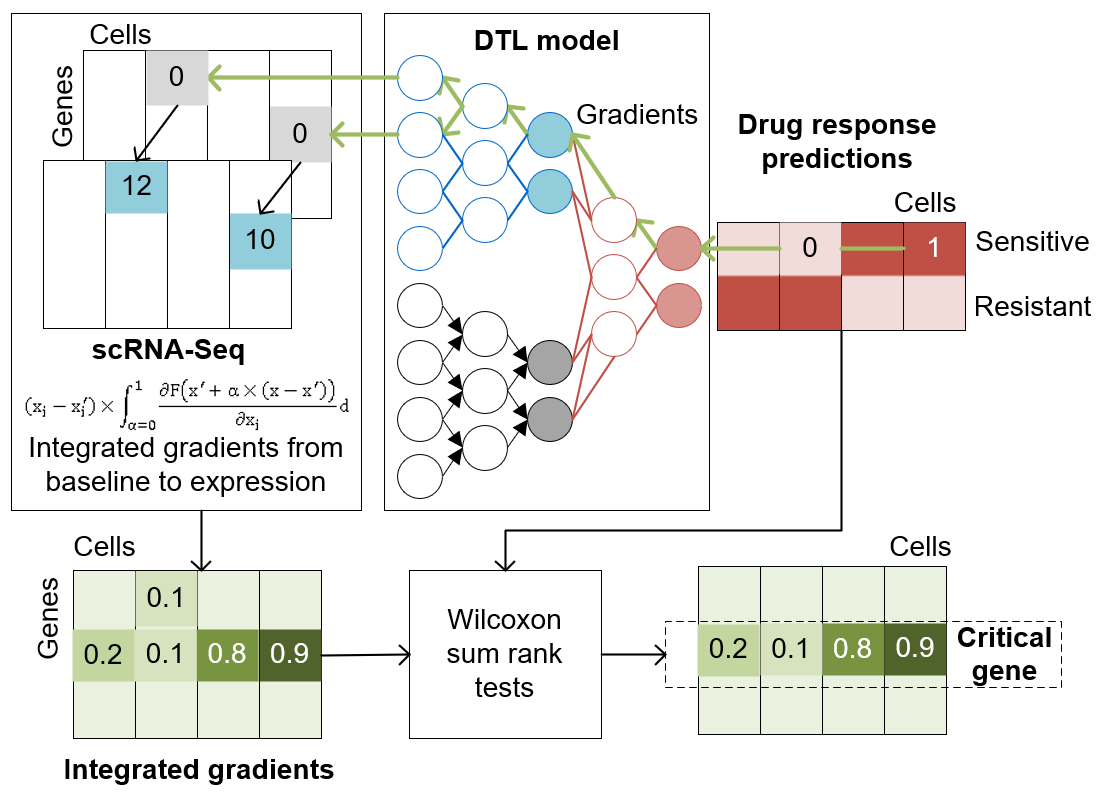


Supplementary Figure S5. Procedure of the integrated gradient method on the gene interpretation from the DTL model. It calculated the importance of the $\mathbf{i}$-th gene expression of the input cell $\mathbf{x}$. $\boldsymbol{\alpha}$ is the scaling coefficient; $\mathbf{x}_{\mathbf{i}}^{\mathbf{'}}$ is the baseline expression level gene $\mathbf{i,}$ which is 0 in our case; and ∂$\mathbf{F}$($\mathbf{x}$) / ∂$\mathbf{x}_{\mathbf{i}}$ represents the gradient of$\mathbf{F}$($\mathbf{x}$) along the $\mathbf{i}$ -th dimension. To select genes that have significantly higher IG values within the sensitive (or resistant) cell cluster, we utilized the Wilcoxon test. We considered the genes with Bonferroni adjusted p-values < 0.05, log-fold changes > 0.1, and the percentage of cells with IG scores in either group higher than 0.2 as CGs.

Supplementary Figure S6. Grid test of 480 parameter combinations across six datasets. We showcased a grid parameter tuning result, including 480 combinations of six hyperparameters (e.g., bulk sampling method, predictor dimension, learning rate, single-cell encoder dimension, dropout, and bottleneck dimension). Each box shows the minimum, first quartile, median, third quartile, and maximum F1 scores. Dots represent outliers. Source data are provided as Source Data 5: grid-search test result of scDEAL on six datasets in terms of F1-score.


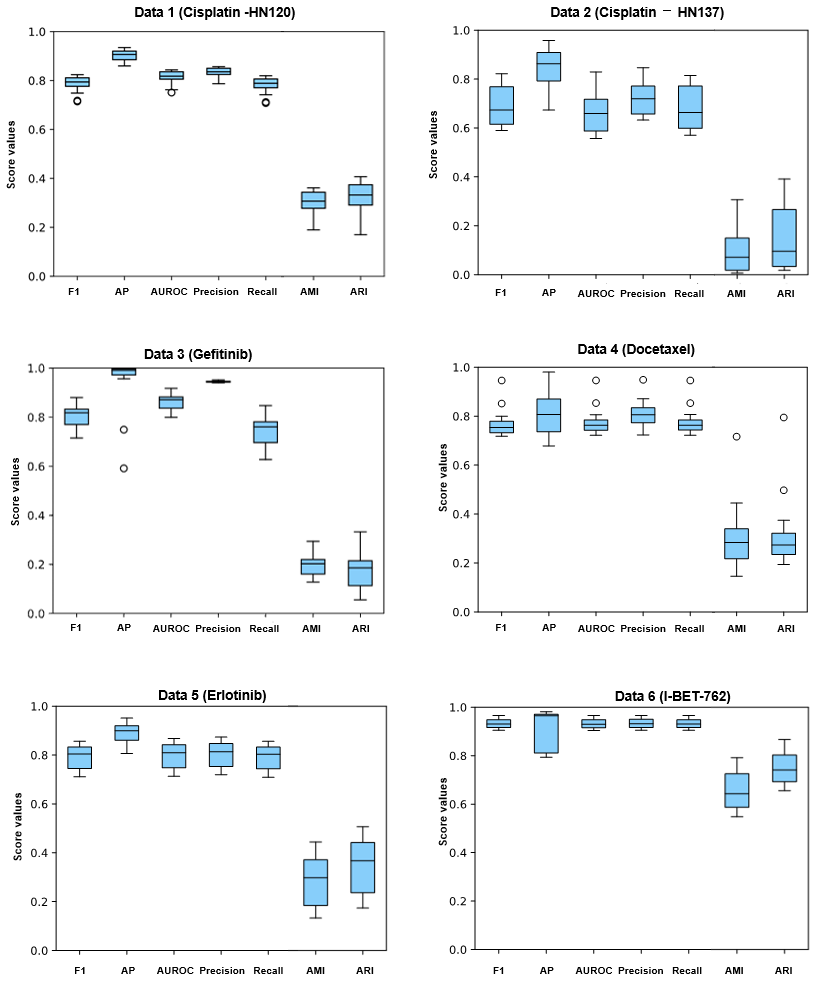


Supplementary Figure S7. Robustness test of scDEAL on six datasets in terms of seven metrics. The experiments were performed using a stratified strategy to repeat the subsampling of 80% cells 20 times. Each box shows the minimum, first quartile, median, third quartile, and maximum F1 scores. Dots represent outliers.


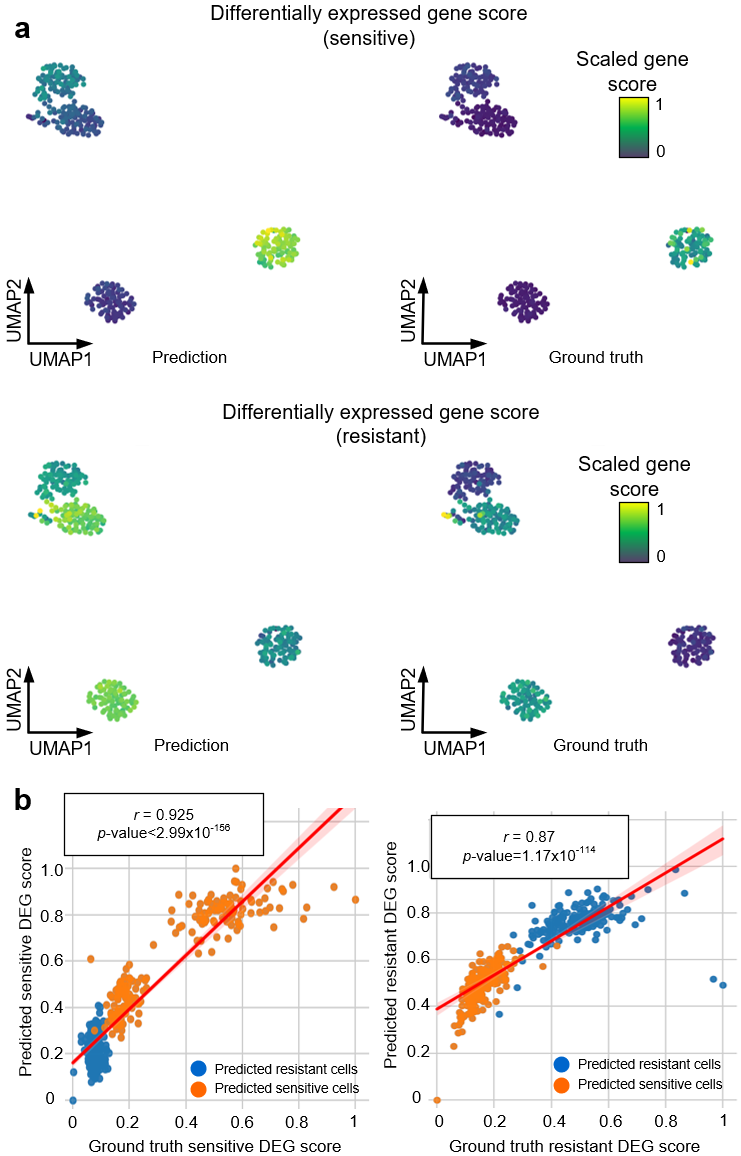


**Supplementary Figure S8.** **Expanded investigation of DEG scores for the case study of Data 1.** (**a**) UMAP plot colored by sensitive (and resistant) gene score derived from differentially expressed genes in the predicted and ground truth sensitive (and resistant) cluster. Source data are provided as Source Data 8: sensitive and resistant DEG scores in predicted and ground truth sensitive and resistance cells in Data 1. (**b**) The plot displays the one-tail Pearson’s correlation test between the gene scores derived from the predicted and the ground-truth cell labels. The error bands showed the 95% confidence interval of the regression. Source data are provided as Source Data 8: sensitive and resistant DEG scores in predicted and ground truth sensitive and resistance cells in Data 1.


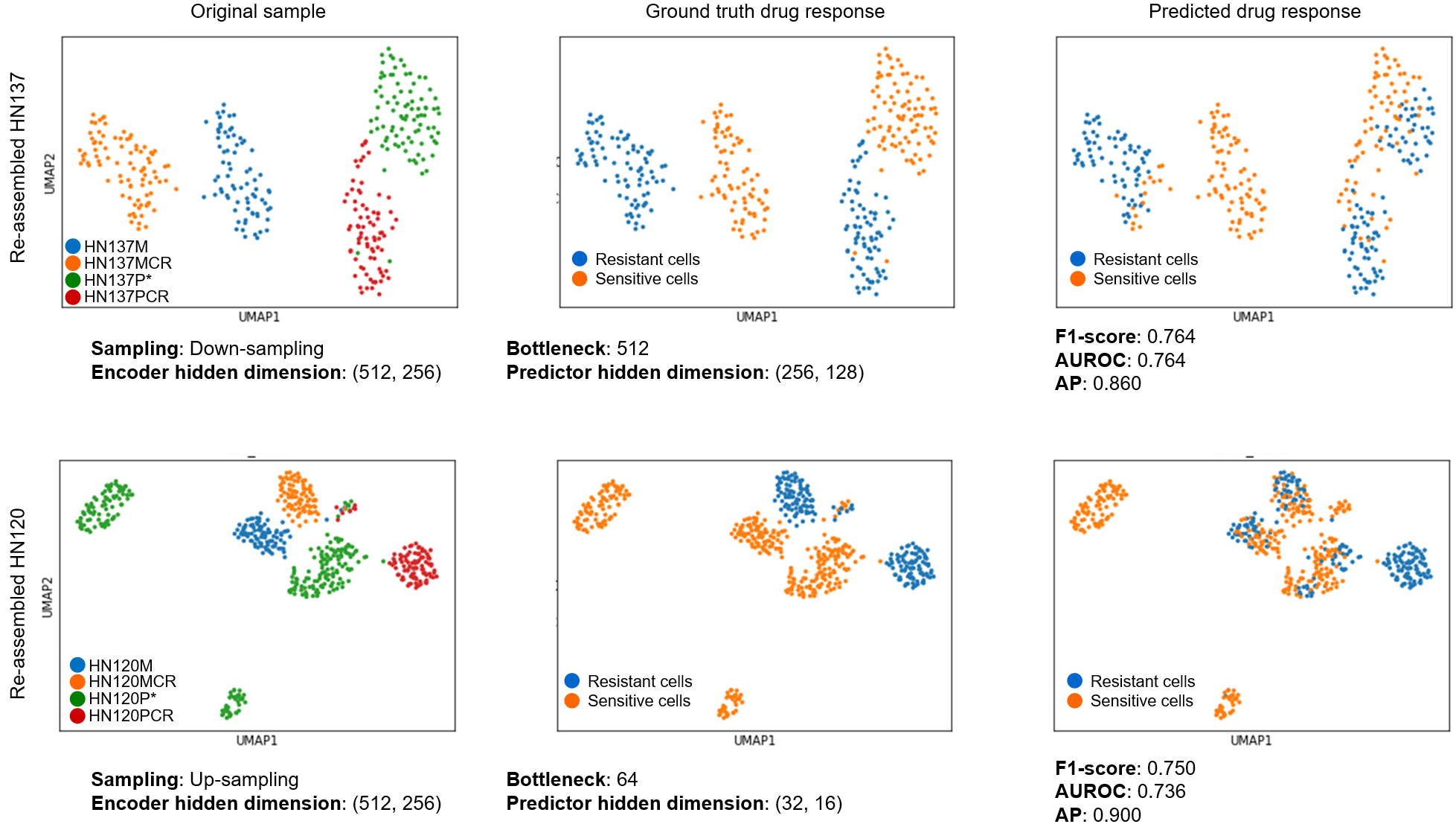


**Supplementary Figure S9**. **scDEAL analysis of re-assembled HN120 and HN137 data.** The scRNA-seq data was re-assembled by switching the HN120P with HN137P in the original datasets (marked with *). UMAPs with sample labels, ground truth drug response, and predicted drug response are showcased. For each analysis, the optimized (based on the grid-search method) parameters and evaluation scores were given below.
