# Supplementary material for: Deep transfer learning of cancer drug responses by integrating bulk and single-cell RNA-seq data
Source: Nat Commun. 2022 Oct 30;13:6494. doi: 10.1038/s41467-022-34277-7 (PMC9618578; doi:10.1038/s41467-022-34277-7)
Supplement: Supplementary file 3 — Description to Additional Supplementary Information [file 41467_2022_34277_MOESM3_ESM.pdf]

Description of Additional Supplementary Information

Supplementary Data 1. Drug-sensitive CGs in the HN120P (sensitive cell group) cells and drug-resistant CGs in the HN120PCR cells.

Supplementary Data 2. Gene Oncology (GO) pathway enrichment analysis of the 868 drug-resistant genes.
