# Supplementary material for: Deep transfer learning of cancer drug responses by integrating bulk and single-cell RNA-seq data
Source: Nat Commun. 2022 Oct 30;13:6494. doi: 10.1038/s41467-022-34277-7 (PMC9618578; doi:10.1038/s41467-022-34277-7)
Supplement: Supplementary file 6 — Reporting Summary [file 41467_2022_34277_MOESM6_ESM.pdf]

nature portfolio

Corresponding author(s): Anjun Ma and Qin Ma

Last updated by author(s): 10/04/2022

Reporting Summary

Nature Portfolio wishes to improve the reproducibility of the work that we publish. This form provides structure for consistency and transparency in reporting. For further information on Nature Portfolio policies, see our Editorial Policies and the Editorial Policy Checklist.

Statistics

For all statistical analyses, confirm that the following items are present in the figure legend, table legend, main text, or Methods section.

n/a	Confirmed	
☐	☒	The exact sample size (*n*) for each experimental group/condition, given as a discrete number and unit of measurement
☐	☒	A statement on whether measurements were taken from distinct samples or whether the same sample was measured repeatedly
☐	☒	The statistical test(s) used AND whether they are one- or two-sided<br>*Only common tests should be described solely by name; describe more complex techniques in the Methods section.*
☒	☐	A description of all covariates tested
☐	☒	A description of any assumptions or corrections, such as tests of normality and adjustment for multiple comparisons
☐	☒	A full description of the statistical parameters including central tendency (e.g. means) or other basic estimates (e.g. regression coefficient) AND variation (e.g. standard deviation) or associated estimates of uncertainty (e.g. confidence intervals)
☐	☒	For null hypothesis testing, the test statistic (e.g. F, t, r) with confidence intervals, effect sizes, degrees of freedom and P value noted<br>*Give P values as exact values whenever suitable.*
☒	☐	For Bayesian analysis, information on the choice of priors and Markov chain Monte Carlo settings
☒	☐	For hierarchical and complex designs, identification of the appropriate level for tests and full reporting of outcomes
☐	☒	Estimates of effect sizes (e.g. Cohen's *d*, Pearson's *r*), indicating how they were calculated

Our web collection on statistics for biologists contains articles on many of the points above.

Software and code

Policy information about availability of computer code

Data collection	Not applicable.
Data analysis	The source code of scDEAL is freely available on (https://github.com/WINGHARE/scDEAL). Other tools and packages used in this paper include: Python version 3.7.10, numpy version 1.18.1, torch version 1.4.0, network version 2.4, pandas version 0.25.3, Monocle version 3, R version 4.1.0, SCANPY version 1.8.1, ClusterProfiler version 3.13.

For manuscripts utilizing custom algorithms or software that are central to the research but not yet described in published literature, software must be made available to editors and reviewers. We strongly encourage code deposition in a community repository (e.g. GitHub). See the Nature Portfolio guidelines for submitting code & software for further information.

Data

Policy information about availability of data

All manuscripts must include a data availability statement. This statement should provide the following information, where applicable:

- Accession codes, unique identifiers, or web links for publicly available datasets
- A description of any restrictions on data availability
- For clinical datasets or third party data, please ensure that the statement adheres to our policy

GDSC is publicly available through the website (https://www.cancerrxgene.org/). Drug response annotation including half maximal inhibitory concentration (IC50) and area under the dose-response curve (AUC) are available through the page https://www.cancerrxgene.org/downloads/bulk_download. Gene expression data (RMA-normalized basal expression profiles) for cell lines can be access on GDSC (https://www.cancerrxgene.org/gdsc1000/GDSC1000_WebResources/Home.html). The CCLE cell line expression profile and PRISM cell line viability assay are available from https://depmap.org/portal/. The six scRNA-seq data used in this study are available in the GEO database without access restrictions under accession codeGSE117872, GSE112274, GSE149383, GSE110894, and GSE140440.

Field-specific reporting

Please select the one below that is the best fit for your research. If you are not sure, read the appropriate sections before making your selection.

☒ Life sciences ☐ Behavioural & social sciences ☐ Ecological, evolutionary & environmental sciences

For a reference copy of the document with all sections, see nature.com/documents/nr-reporting-summary-flat.pdf

Life sciences study design

All studies must disclose on these points even when the disclosure is negative.

Sample size	No data collection was involved in the present study. We analyzed 6 publicly available single-cell RNA-seq datasets. These 6 datasets represent all the available cancer data treated by different drugs. No statistical methods were used to predetermine the sample size or the number of datasets.
Data exclusions	All cells and genes of each single-cell data set were used; and no exclusion was done prior to analysis.
Replication	We did not perform biological or technical replications. Conflating technical and biological variability are not included. All data are collected from public domain.
Randomization	Randomization is not relevant to this study since each data was analyzed separately. The analyses reported in this paper do not involve between sample comparison.
Blinding	Blinding is not relevant since no data collection was involved in the present study.

Reporting for specific materials, systems and methods

We require information from authors about some types of materials, experimental systems and methods used in many studies. Here, indicate whether each material, system or method listed is relevant to your study. If you are not sure if a list item applies to your research, read the appropriate section before selecting a response.

Materials & experimental systems

n/a	Involved in the study	
☒	☐	Antibodies
☒	☐	Eukaryotic cell lines
☒	☐	Palaeontology and archaeology
☒	☐	Animals and other organisms
☒	☐	Human research participants
☒	☐	Clinical data
☒	☐	Dual use research of concern

Methods

n/a	Involved in the study	
☒	☐	ChIP-seq
☒	☐	Flow cytometry
☒	☐	MRI-based neuroimaging
